# Characterization and phylogenetic analysis of the complete mitochondrial genome of *Rhinogobius wuyanlingensis* (Gobiiformes: Gobiidae: Gobionellinae)

**DOI:** 10.1080/23802359.2022.2097488

**Published:** 2022-07-22

**Authors:** Lin Song, Xiao Jiang Chen, Huang Xin Mao, Quan Wang

**Affiliations:** Jiangsu Agri-animal Husbandry Vocational College, Taizhou, Jiangsu, China

**Keywords:** Mitochondrial genome, *Rhinogobius wuyanlingensis*, Gobionellinae, phylogenetic

## Abstract

*Rhinogobius wuyanlingensis* is endemic to the upper reaches of Feiyunjiang river basin. In this article, the complete mitochondrial genome (mtDNA) for this freshwater goby was first determined. Sequence analysis showed the genome consisted of 13 protein-coding genes, 22 tRNA genes, two ribosomal RNA genes, and two main non-coding regions (the control region and the origin of the light strand replication). This circular molecule was 16,491 bp in length with a slight AT bias of 53.5%. In the phylogenetic tree, *R. wuyanlingensis* was closer to *Rhinogobius brunneus*, *Rhinogobius yonezawai, Rhinogobius flumineus*, and *Rhinogobius cliffordpopei*. The mitochondrial genome of *R. wuyanlingensis* reported here would provide basal molecular data for evolution, taxonomy, and population genetics of *Rhinogobius*.

*Rhinogobius* is a genus of freshwater goby rather common over several islands of the West Pacific and continental Asia (Huang et al. 2016). Because of the high isolation of habitats, *Rhinogobius* is a species-rich genus of about 129 valid species according to Fishbase statistic. *Rhinogobius wuyanlingensis* (Yang et al. 2008) can be served as a pint-size ornamental fish known from upper tributary of Feiyunjiang basin, Zhejiang. Although this species is similar to *Rhinogobius lindbergi* in having preopercular canal with two terminal pores as other species with three pores or lacking peropercular, it can be distinguished from the morphology perspective by P rays 17–18 (*vs.* 19–20), A rays I, 8 (*vs.* modally I, 9), and PreD 7–9 (*vs.* 0) (Yang et al. 2008). Here, we sequenced the mtDNA of *R. wuyanlingensis* to provide useful basal information for genome evolution and species phylogeny studies of *Rhinogobius*, and it may also facilitate population genetics research of Gobionellinae in the future.

*R. wuyanlingensis* samples were collected from Wuyanling National Natural Conservation Area, Taishun County, Zhejiang Province, China (27°71′53.15″ N, 119°67′10.21″E). The specimen were stored in 95% ethanol at 4 °C in Aquatic Science and Technology Institution Herbarium (https://www.jsahvc.edu.cn/, Voucher number ASTIH-21b1108d21, The person in charge of the collection: Lin Song, Email: tianxinlinger@126.com). We used Tguide Cell/tissue genomic DNA Extraction Kit (OSR-M401) (Tiangen, Beijing, China) to extract the total genomic DNA from muscle after transported to Shanghai Genesky Biotechnologies Inc. There followed the steps of sample quality control, DNA library construction, PCR amplification, size selection, library quality check and library pooling. Qualified PCR products were sequenced on Illumina HiSeq 4000 Sequencing platform (Illumina, CA, USA). MetaSPAdes (Nurk et al. 2017) was used to assemble the fragments sequences. Subsequently, the whole mitochondrial genome was annotated through MitoMaker (Bernt et al. 2013) and then submitted to GenBank with accession number OM617722.

The final assembly mitogenome was a 16,491 bp circular molecule, with the nucleotide composition of 27.5% A, 26% T, 16.8% G, 29.7% C, displaying a slight AT-rich feature (53.5%), which was similar to other *Rhinogobius* fishes (Xie et al. 2015). The sequence of *R. wuyanlingensis* revealed the typical gene content: 13 typical protein-coding genes (PCGs), 22 tRNA genes, two rRNA (12SrRNA and 16S rRNA) genes, and two main non-coding regions (control region and light strand origin of replication). The pattern on structural organization of *R. wuyanlingensis* complete mtDNA was substantially in accordance with other Gobies (Wang et al. 2019). Besides ND6 and eight tRNA genes *(tRNA^Gln^*, *tRNA^Ala^*, *tRNA^Asn^*, *tRNA^Cys^*, *tRNA^Tyr^*, *tRNA^Ser(UCN)^*, *tRNA^Glu^*, and *tRNA^Pro^*) encoded on light strand (L-strand), all other genes were encoded on the heavy strand (H-strand). Most of the 13 PCGs initiated with the standard ATG except for COI GTG with the orthodox start codon. Termination codons were varied with complete TAA (*ND1*, *ND2*, *COI*, *ATP8*, *ATP6*, and *ND4L*), TAG (*ND3*, *ND5*, and *ND6*) or incomplete TA (*COIII*), T (*COII*, *ND4*, and *Cytb*). The longest protein-coding gene was *ND5* (1839 bp), whereas the shortest was *ATP8* (165 bp). The sequence overlaps among PCGs was found between *ATP8-ATP6*, *ATP6-COIII*, *ND4L-ND4*, *ND5-ND6*, and they overlapped 7, 1, 7, and 4 bp, respectively. Twenty-two tRNA genes ranged in size from 66 to 76 bp were distributed through the whole mitogenome. Two ribosomal RNA genes (12s rRNAs and 16s rRNAs) were divided by *tRNA^Val^* with the length of 951 and 1656 bp separately. The control region located between the *tRNA^Pro^* and *tRNA^Phe^* was 474 bp, while the origin of the light strand replication extended up to 30 nucleotides.

Altogether, a set of 24 Gobionellinae PCG sequences was downloaded from GenBank to validate the phylogenetic position of *R. wuyanlingensis*, which were firstly aligned on MEGA X. Then the most appropriate evolutionary model to the data was estimated. Finally, Maximum likelihood (ML) analysis was performed with 1000 bootstrap replicates under mtREV + G + I + F model choosing *Pandaka pygmaea* as outgroup (Kumar et al. 2018). In Figure 1, *Rhinogobius* firstly joined with genera *Gymnogobius*, *Luciogobius*, and next clustered with *Eugnathogobius*, *Mugilogobius* into a clade. Then, all these species together with *Acanthogobius* constituted a clade. Finally, this clade formed sister groups with *Brachygobius*. On the other hand, there were two sister clades inside the genus *Rhinogobius*, clade I combined *Rhinogobius brunneus*, *Rhinogobius yonezawai*, *Rhinogobius flumineus*, *Rhinogobius cliffordpopei* (Zhong et al. 2018), and *R. wuyanlingensis* while *Rhinogobius estrellae*, *Rhinogobius tandikan*, *Rhinogobius giurinus* (Xie et al. 2015), and *Rhinogobius similis* formed clade II. Furthermore, *R. wuyanlingensis* was most related to *R. brunneus*, *R. yonezawai*, *R. flumineus*, and *R. cliffordpopei*. We expect these results would provide an important basis for future phylogenetic relationship investigations of Gobionellinae as well as evolutionary analysis in family Gobiidae.

**Figure 1.**
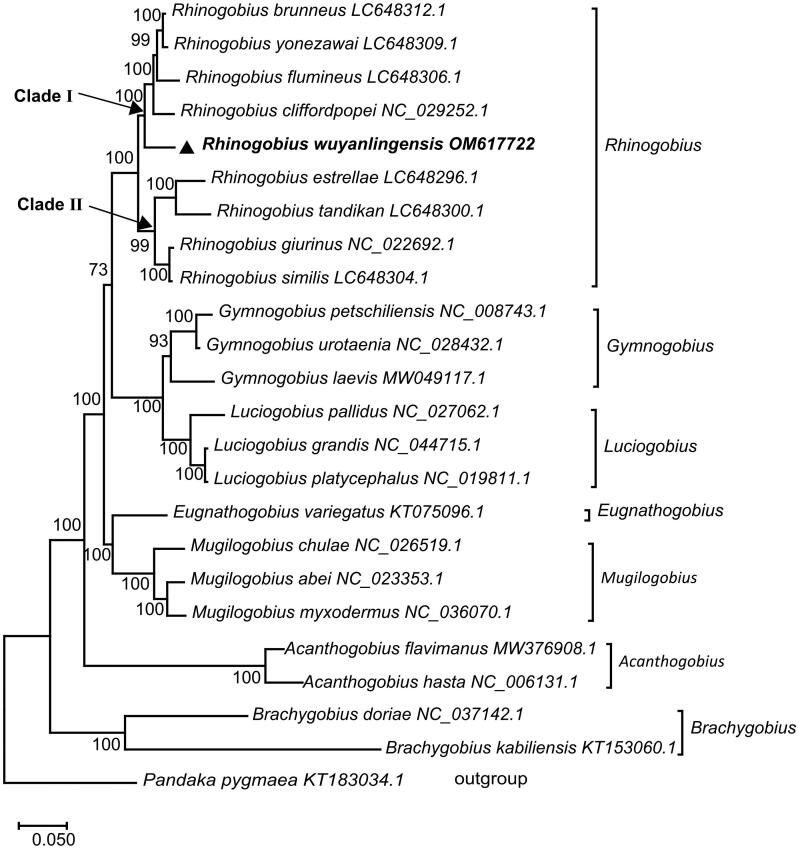
Maximum-likelihood (ML) phylogenetic tree reconstructed using concatenated mitochondrial protein-coding genes of *R. wuyanlingensis* and other 23 fishes. Accession numbers were indicated after the species names. The tree topology was evaluated by 1000 bootstrap replicates. Bootstrap values at the nodes correspond to the support values for ML methods.

## Data Availability

The genome sequence data that support the findings of this study are openly available in GenBank of NCBI at https://www.ncbi.nlm.nih.gov/ under the reference number OM617722.1. The associated ‘BioProject,’ ‘Bio-Sample,’ and ‘SRA’ numbers are PRJNA807060, SAMN25949221, and SRR18030029, respectively.
